# An early seizure variant type of a male Rett syndrome patient with a *MECP2* p.Arg133His missense mutation

**DOI:** 10.1002/mgg3.532

**Published:** 2018-12-19

**Authors:** Jin A. Yoon, Yongjin Yoo, Je Sang Lee, Young Mi Kim, Yong Beom Shin

**Affiliations:** ^1^ Department of Rehabilitation Medicine Pusan National University School of Medicine and Biomedical Research Institute, Pusan National University Hospital Busan Korea; ^2^ Department of Biomedical Sciences Seoul National University College of Medicine Seoul Korea; ^3^ Department of Pediatrics Pusan National University School of Medicine and Biomedical Research Institute, Pusan National University Hospital Busan Korea

**Keywords:** MECP2 mutation, Rett syndrome, whole exome sequencing

## Abstract

**Background:**

The clinical spectrum of Rett syndrome (RTT; Mendelian Inheritance in Man [MIM] #312750) in males is considered to be wider than previously expected. Therefore, the existence of RTT with a normal male karyotype is still controversial. Here, we report the first case of a male patient presenting with an early seizure type of Rett‐like phenotypes with a missense variant of *MECP2*.

**Method:**

An 8‐month‐old male was admitted to the pediatric department due to an initial seizure event following aspiration pneumonia and was referred to our clinic for the evaluation of unexplained neuroregression. Genomic DNA was prepared from venous blood by standard procedures and was processed at the Yale Center for Genome Analysis (YCGA) for whole exome sequencing (WES). Processing of sequence data, variant calling, and the identification of de novo mutations were then performed. Direct Sanger sequencing was performed following PCR amplification.

**Result:**

In this patient with a normal karyotype, WES analysis led to the identification of a novel, de novo missense variant of MECP2 (p.Arg133His) that is not observed in the normal population.

**Conclusion:**

This rare case of an p.Arg133His hemizygous *MECP2* missense mutation could guide future treatment and follow‐up plans for RETT‐like phenotypes.

## 1 INTRODUCTION

Rett syndrome (RTT; Mendelian Inheritance in Man [MIM] #312750), first described in 1966 by Andreas Rett, is a neurodevelopmental disorder affecting postnatal brain growth that is mostly recognized in girls with a prevalence of 1 in 10,000–15,000 births (Laurvick et al., 2006; Rett, 1966). Most cases are sporadic with rare familial cases reported (Amir et al., 1999; Schanen et al., 1997). In 1985, Hagberg first set up and described clinical diagnostic criteria and the definition of RTT including significant regression in abilities and their typical features. Subsequently, clinical symptoms comprised the only diagnostic tool until mutations in the gene encoding methyl‐CpG binding protein 2 (MECP2) were first identified in 1999 by Amir et al. (1999). MECP2 mutations reportedly occur in 95%–97% of females with RTT based on recent mutation detection assays (Neul et al., 2008). Total 20 cases of mutation in the *MECP2* gene in a male was reported, most recently by Shimada et al. (2013). Clinical spectrum of *MECP2* mutations in males is considered to be wider than expected (Reichow, George‐Puskar, Lutz, Smith, & Volkmar, 2015). Both clinical symptoms and specific genetic mutations might modulate disease severity. Otherwise, previous research regarding the dependence of diverse phenotypes on different genotypes is not applicable for male Rett patients (Cheadle et al., 2000; Hoffbuhr, Moses, Jerdonek, Naidu, & Hoffman, 2002). Therefore, the existence of RTT with a normal male karyotype is still controversial.

There have been few reports of mutational analyses in males with normal karyotypes and loss‐of‐function MECP2 mutations in different countries including Korea. Here, we report the rare case of an R133H missense hemizygous MECP mutation.

### 1.1 Ethical compliance

Approval of this study was obtained from the Institutional Review Board of our hospital.

## 2 CASE REPORT

The 8‐month‐old boy was born at term without any unusual birth history (38 weeks, 3,150 g, by Cesarean delivery) to a 45‐year‐old father and 36‐year‐old mother. He had one brother (12‐year‐old) and sister (8‐year‐old). None of the family members had any medical history during the growth period.

He was admitted to the pediatric department due to an initial seizure event following aspiration pneumonia and was referred to our clinic for the evaluation of unexplained neuroregression. Although he was hypotonic from birth, he achieved a social smile at 3 months and started head control during the first 4 months. He rolled over, and nearly grasped his toys with prone position at 6 months. Generalized tonic–clonic type seizures at 6 months were his first clinical symptom, a detailed history revealed delays in developmental milestones after that. Electroencephalogram (EEG) findings showed abnormal awake and sleep recordings due to slow background activity, suggestive of diffuse cerebral dysfunction with symptomatic or cryptogenic seizures. Magnetic resonance imaging showed cerebral hypoplasia especially in the frontal and temporal lobes at approximately 4 years of age. He was observed at the outpatient clinic for developmental delays associated with encephalopathy and seizure events, which occurred hundreds of times for 2 years and were fairly well‐controlled with valproic acid, phenobarbital, and clonazepam.

At 26 months after surgery for bilateral cryptorchidism, progressive respiratory difficulty persisted and weaning from the ventilator was not possible; repetitive aspiration pneumonia occurred as he was unable to proceed with sputum expectoration. Therefore, tracheostomy was performed and night‐time breathing using a ventilator was maintained subsequently. At the time of admission, repetitive hand flipping without purpose and lip smacking was observed during examination, although epileptiform discharges were not observed during EEG, we decided to proceed with additional evaluation other than that previously considered at this point. The various clinical features of the patient are described in Table 1.

**Table 1:** Scoring of various clinical features of the patient by revised diagnostic criteria of RTT

Clinical symptom	Description
A period of regression followed by recovery or stabilization	Yes
Main criteria	
Partial or complete loss of acquired purposeful hand skills	Indefinite
Partial or complete loss of acquired spoken language	Not obtained
Gail abnormalities: Impaired or absence of ability	Not obtained
Stereotypic hand movement	Hand wringing without purpose
Supportive criteria for atypical RTT	
Breathing disturbance when awake	Ventilator breathing from 26 months
Bruxism when awake	No
Impaired sleep pattern	Yes
Abnormal muscle tone	Hypotonia from birth
Peripheral vasomotor disturbances	No
Scoliosis/kyphosis	Thoracolumbar scoliosis
Growth retardation	Yes
Small cold hands and feet	Yes
Inappropriate laughing/screaming spells	Yes
Diminished response to pain	Yes
Intense eye communication—“eye pointing”	No

There were no abnormal findings based on laboratory investigation, and genetic analysis of mutations including Prader‐Willi gene, spinal muscular atrophy gene, and other chromosomal aberrations. Chromosome analysis revealed a 46, XY karyotype. A muscle biopsy also demonstrated no abnormal findings.

## 3 WHOLE EXOME SEQUENCING AND SANGER VALIDATION

This study was reviewed by the appropriate ethics committee. Blood samples from the patient and parents were obtained using standard protocols after receiving informed written consent. Genomic DNA was prepared from venous blood using standard procedures and was processed at Yale Center for Genome Analysis (YCGA) for whole exome sequencing (WES) as described previously (Choi et al., 2009). The processing of sequence data, variant calling, and identification of de novo mutations were performed as described previously (Zaidi et al., 2013). Direct Sanger sequencing was performed using standard methods following PCR amplification.

## 4 RESULTS

**Figure 1:**
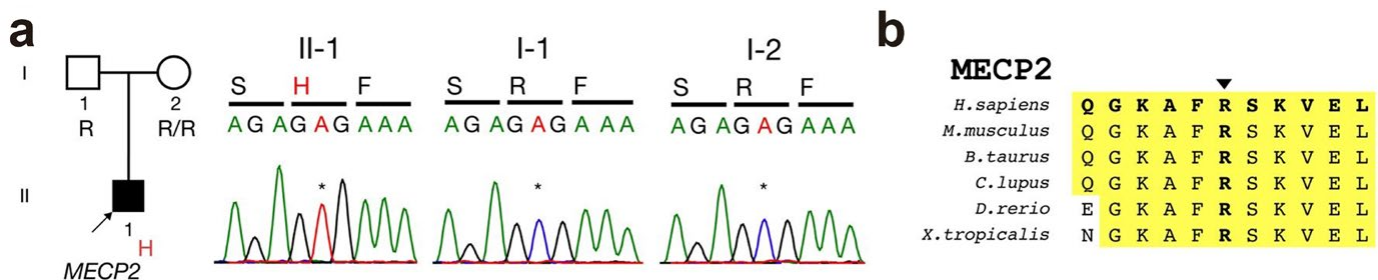
(a) Pedigrees of the families and Sanger traces confirming the de novo variants in *MECP2* specific to the patients. (b) Conservation of the Arg133 residue in orthologs from different vertebrate species

**Figure 2:**
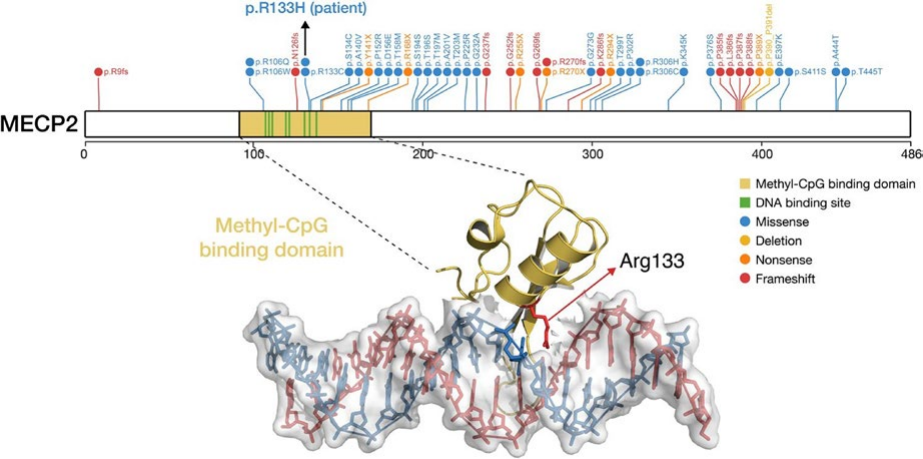
The variant from patient was found in the Methyl‐CpG binding domain (PDB ID: 5BT2; Chia et al., 2016). High frequency of *MECP2* variants (frequency ≥10) from RettBASE (Christodoulou, Grimm, Maher, & Bennetts, 2003) were indicated. *B. Taurus*: *Bos taurus* (cow); *C. lupus*: *Canis lupus* (wolf); *H. sapiens*: *Homo sapiens* (human); *M. musculus*: *Mus musculus* (mouse); *X. tropicalis*: *Xenopus tropicalis* (frog)

To understand the genetic basis of the clinical features according to RTT diagnosis criteria (Table 1), we performed WES analysis of the patient and his healthy parents. WES data were of high quality (Table 2) and de novo, rare recessive variants, including homozygous, hemizygous, and compound heterozygous mutations, were identified. Patient‐specific variants were evaluated based on the following criteria: variants should be either loss‐of‐function or nonsynonymous mutations, resulting in amino acid changes in well‐conserved residues across vertebrate species and not present in 1,000 Genomes and ExAC databases. A missense variant of *MECP2 *(encoding methyl‐CpG binding protein 2; specifically, p.Arg133His; Figure 1a) was not found in the 1,000 Genomes or ExAC database. The mutated amino acid residue (p.Arg113) was highly conserved among vertebrate orthologs (Figure 1b) and was located in the methyl‐CpG binding domain, which is involved in transcriptional repression by this complex (Figure 2; Clouaire & Stancheva, 2008; Du, Luu, Stirzaker, & Clark, 2015). There was no other homozygous, compound heterozygous mutation but only de novo mutation in MECP2 (Table 3; Adzhubei et al., 2010; Ng & Henikoff, 2003; Pollard, Hubisz, & Siepel, 2010).

**Table 2:** Basic statistics of the whole exome sequencing runs

	Proband	Healthy mother	Healthy father
Read length (bp)		74	
Number of reads (*M*)	77.6	63.0	63.4
Mean coverage (*X*)	74.6	61.3	61.7
% of targeted bases read at least 8×	95.5	94.5	94.4
Per‐base error rate (%)	0.46	0.44	0.43

**Table 3:** List of notable mutations from patient

Gene	Chromosomal position (hg19)	Nucleotide substitution	Zygosity	Coverage (ref. cov./total cov.)	Impact on protein	Amino acid change	Amino acid location/protein length	*PhyloP*	*SIFT*	*PolyPhen‐2*
*MECP2*	chrX:153296881	C>T	Hemizygous	0/37	Missense	p.Arg133His	133/486	5.44	0.00	1.00

## 5 DISCUSSION

We report the first case of a male patient with an early seizure type, Rett‐like phenotype that is associated with a missense variant of *MECP2 *(p.Arg133His). Abrogation of MECP2 might affect all cells of the male patient and it could lead to the presentation of RTT‐like phenotypes including early‐onset seizure and breathing problems. Unlike typical RTT with commonly known clinical symptoms in girls, young males with severe progressive developmental difficulty should be considered as a variant form of RTT as part of the differential diagnosis. In early‐onset seizure variant of RTT, distinct phenotypes including early seizure variant with early seizure onset prior to regression may present like our patient (Artuso et al., 2010).

**Figure 3:**
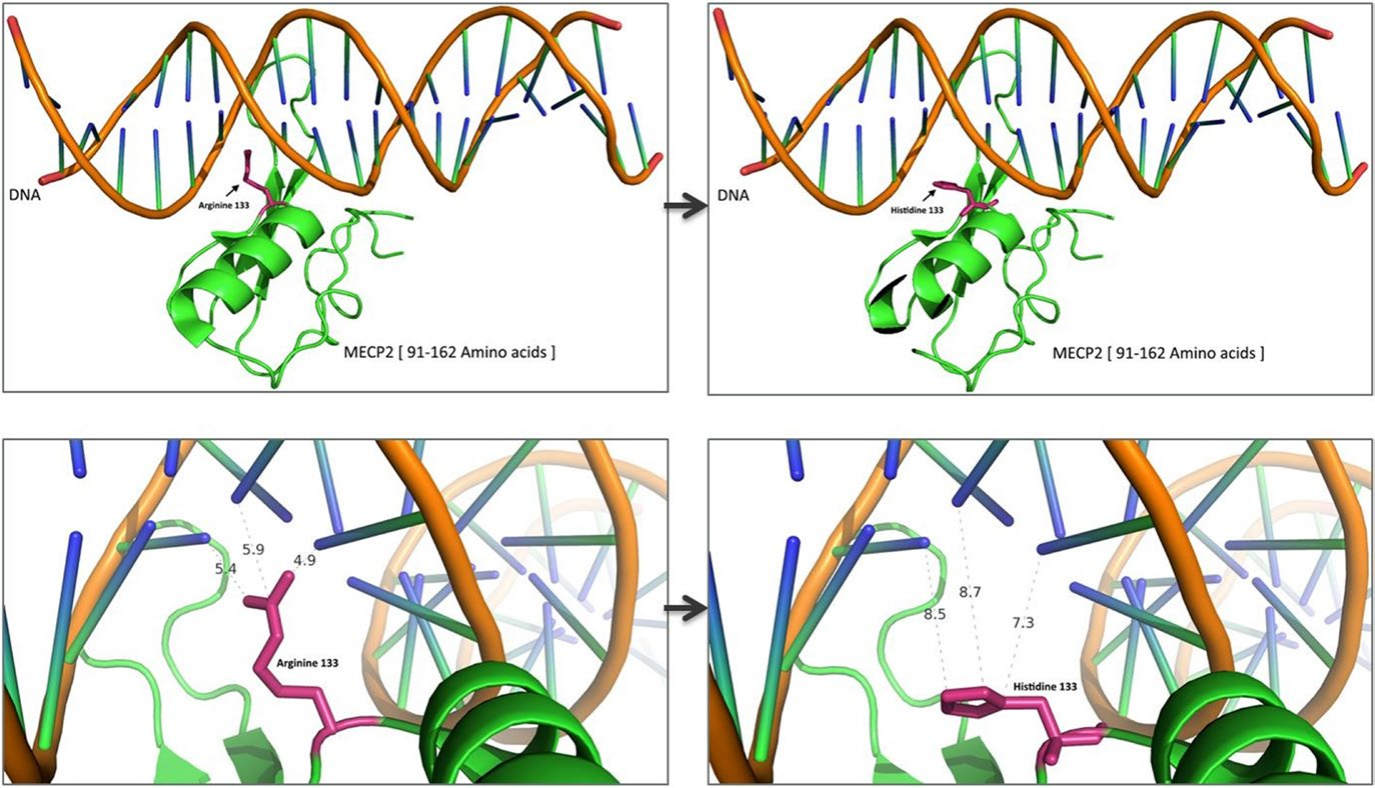
In R133H variant, the gene expression process could be explained as the ring‐shaped histidine might have had problem with less affinity compared to Y‐shaped arginine both in shape and distance during the RNA transcription process

In this patient who had a normal karyotype, WES analysis led to the identification of a novel, de novo missense variant of *MECP2* (p.Arg133His), which was not observed in the normal population. Interestingly, several studies have reported other amino acid changes in MECP2 (p.Arg133Cys; frequency = 217; % in DB = 4.6) at the same residue, but few male cases with same mutation (p.Arg133His; frequency = 8; % in DB = 0.2) have been reported in RettBASS (http://mecp2.chw.edu.au). In our case, which was associated with an R133H variant, gene expression could be affected as the ring‐shaped histidine might interfere with the protein function, resulting in less affinity, as compared to that with the Y‐shaped arginine during the RNA transcription process (Figure 3).

Recently, the clinical spectrum of MECP2 mutations in males was considered to be wider than expected (Reichow et al., 2015). Previously, *MECP2* gene mutations were considered lethal for male patients (Choi et al., 2009; Trevathan, 1989). MECP2 acts as a transcriptional repressor in all cells of the body, including those of the brain. It is found at high concentrations in neurons and plays a critical role in brain development during the perinatal period (Luikenhuis, Giacometti, Beard, & Jaenisch, 2004). As males have one *MECP2* gene located on their X chromosome, mutations in the only copy could affect all cells in the body, and these cases can present with early‐onset seizure and breathing problems, as observed with our patient. The diagnosis of RTT is still based on clinical presentation. As described in the revised diagnostic criteria and nomenclature of RTT in 2010 (Neul et al., 2010), patients that harbor mutations in *MECP2* but do not meet the clinical criteria for RTT are considered to have an MECP2‐related disorder. A certain form of neuroregression is required for the diagnosis of RTT, even atypical types. Although our patients displayed normal psychomotor development throughout the first 6 months, he showed subsequent neuroregression since then which is required for the diagnosis of any type of RTT. He was compatible with the early seizure variant type of atypical RTT (Hanefeld, 1985), satisfying three or four main criteria and eight of ten supportive criteria.

As recent systemic review describing 32 cases of RTT in males (Reichow et al., 2015), the connection between genotype and phenotype to explain the specific clinical presentation of different MECP2 mutations in RTT is still obscure. Yet, there are no specific treatment options according to types of mutation in Rett syndrome. Potential approach to turn off the mutant allele and activating the normal allele by detecting the locus of de novo MECP2 mutations has been discussed for potential treatment options (Chapleau et al, 2013). Understanding the dysfunction of MeCP2 mutation and identifying related dysregulated genes which contribute to specific phenotype will be helpful to understand the molecular mechanism, finally providing clues to potential treatment. A previous study correlating genotype and phenotype in 116 patients reported an R133H missense mutation of MECP2 from a G398A transition in a girl with atypical RTT who was ambulatory, with no clear period of regression, and with autistic behavior (Hoffbuhr et al., 2001). A case of a boy (normal XY karyotype) with an R133H missense mutation in MECP2 was also reported, and he was classified as classic RTT as he fulfilled eight of nine clinical criteria (Armstrong, Pineda, Aibar, Geán, & Monrós, 2001). Although this report contained insufficient information regarding the patient’s clinical symptoms, it bears no resemblance with our patient who presented with the early seizure onset with progressive respiratory difficulty.

MECP2 mutations are localized to two of four functional methyl‐CpG‐binding domains (MBDs) and transcriptional repression domains (TRDs), which deacetylase histone in DNA to inactivate the chromosome. Similar to that concluded previously by Hoffbuhr et al. (2001), our patient with a missense mutation in an MBD could be severely affected, as compared to patients with classic type RTT with missense and nonsense mutations in TRDs and C terminal segments.

## 6 CONCLUSION

The existence of RTT‐like symptoms with a normal male karyotype is still controversial; thus, it is important to report information regarding the rare genotypes of male RTT patients in publications. This rare case of an R133H missense mutation in a patient who was hemizygous for MECP2 could help to guide future treatment and follow‐up plans for this condition.

## CONFLICT OF INTEREST

None.
